# Clinical genetics evaluation and testing of connective tissue disorders: a cross-sectional study

**DOI:** 10.1186/s12920-022-01321-w

**Published:** 2022-08-02

**Authors:** Olivia J. Veatch, Jacob Steinle, Waheeda A. Hossain, Merlin G. Butler

**Affiliations:** 1 Department of Psychiatry and Behavioral Sciences, University of Kansas Medical Center, 3901 Rainbow Blvd., MS-4015, Kansas City, KS 66160 USA; 2 Department of Molecular and Integrative Physiology, University of Kansas Medical Center, Kansas City, KS USA; 3 Department of Pediatrics, Medical Center, University of Kansas, Kansas City, KS USA

**Keywords:** Heritable connective tissue disorders, Ehlers–Danlos syndrome, Next-generation sequencing, Phenotype-genotype relationships, Gene variants, Latent class analysis

## Abstract

**Background:**

Heritable connective tissue disorders (HCTDs) consist of heterogeneous syndromes. The diagnosis of HCTDs is aided by genomic biotechnologies (e.g., next-generation sequencing panels) facilitating the discovery of novel variants causing disease.

**Methods:**

Detailed clinical exam data and CLIA-approved genetic testing results from next generation sequencing of 74 genes known to play a role in HCTDs were manually reviewed and analyzed in one hundred consecutive, unrelated patients with phenotypic features indicative of a HCTD referred over a 3.5-year period (2016–2020) to a specialized academic genetics clinic. The prevalence of symptoms was evaluated in the context of genetic variants. We also determined if symptoms among different organ systems were related and performed latent class analysis to identify distinct groups of patients based on symptomatology.

**Results:**

In the cohort of 100 consecutive, unrelated individuals there were four pathogenic, six likely pathogenic and 35 classified potentially pathogenic variants of unknown clinical significance. Patients with potentially pathogenic variants exhibited similar symptom profiles when compared to patients with pathogenic/likely pathogenic variants in the same genes. Although results did not meet a multiple testing corrected threshold, patients with connective tissue symptoms had suggestive evidence of increased odds of having skin (odds ratio 2.18, 95% confidence interval 1.12 to 4.24) and eye symptoms (odds ratio 1.89, 95% confidence interval 0.98 to 3.66) requiring further studies. The best performing latent class analysis results were identified when dividing the dataset into three distinct groups based on age, gender and presence or absence of symptoms in the skeletal, connective tissue, nervous, gastrointestinal and cardiovascular systems. These distinct classes of patients included individuals with: (1) minimal skeletal symptoms, (2) more skeletal but fewer connective tissue, nervous or gastrointestinal symptoms and (3) more nervous system symptoms.

**Conclusions:**

We used novel approaches to characterize phenotype-genotype relationships, including pinpointing potentially pathogenic variants, and detecting unique symptom profiles in patients with features of HCTDs. This study may guide future diagnosis and disease/organ system monitoring with continued improvement and surveillance by clinicians for patients and their families.

**Supplementary Information:**

The online version contains supplementary material available at 10.1186/s12920-022-01321-w.

## Background

Heritable connective tissue disorders (HCTDs) are a diverse group of conditions that encompass symptoms indicative of defects in the organization and synthesis of extracellular matrix components involving many organs [1]. Features often observed in HCTDs or grouped into seven clinical categories including ectopia lentis, scoliosis, chest deformities, stretchable skin, poor healing, joint instability and pain, risk for fractures and skeletal anomalies, gastrointestinal and cardiovascular problems, cerebral aneurysms, and aortic dissections [2–4]. HCTDs include various syndromes with known genetic causes like Marfan syndrome [5], osteogenesis imperfecta [6], Melnick-Needles syndrome [7], cutis laxa [8], thoracic aortic aneurysms [9], and Ehlers–Danlos syndrome (EDS) [10]. Continued advancements in next-generation sequencing (NGS) over the past decade have facilitated the discovery of numerous genetic variants implicated in a variety of HCTDs [11–13]. For example, the genetic basis of 13 reported subtypes of EDS—classical (OMIM: 130000, 130010), classical-like (OMIM: 606408), cardiac-valvular (OMIM: 225320), vascular (OMIM: 130050), hypermobile (OMIM: 130020), arthrochalasia (OMIM: 130060), Dermatosparaxic (OMIM: 225410), kyphoscoliotic (OMIM: 225400, 614557), brittle cornea syndrome (OMIM: 229200, 614170), spondylodysplastic (OMIM: 130070), musculocontractural (OMIM: 601776), myopathic (OMIM: 616471), and periodontal (OMIM: 130080)—has been linked to dominant and recessive variation in 19 genes with only hypermobile EDS having an unresolved genetic basis and considered a diagnosis of exclusion [10, 14]. Consequently, the use of NGS connective tissue gene panels and deletions/duplications including the sequencing of the exome has become increasingly prevalent as a means to refine, differentiate and confirm clinically suspected HCTDs [15]. A growing role for genomic sequencing beyond the exome is also under-consideration with advanced technology and less costly approaches at the gene level for NGS connective tissue gene panels that currently contain upwards of 80 genes with recognized pathogenic variants for HCTDs. Identifying variants that are likely causal for HCTDs can help clinicians better mitigate and manage complications, some life-threatening such as aortic aneurysms and offer testing for at risk family members with genetic consultation as several HCTDs are inherited as autosomal dominant with a 50% recurrence risk for first degree relatives.

Though the accuracy of diagnosing these disorders has increased dramatically using NGS and connective tissue gene panels, patients present with variable expressivity of clinical signs and symptoms that often overlap among genetically distinct HCTDs [14, 16]. It remains unclear if presence of symptoms in specific organ systems are informative to help confirm a particular HCTD diagnosis in lieu of genetic testing. For instance, Marfan syndrome, EDS, and Loeys-Dietz syndrome are each influenced by variation in different genes yet have clinical overlap regarding cardiovascular, skeletal, craniofacial, ocular, and cutaneous features [4]. Given this information, although clinicians may rely on certain clinical features that are hallmarks of HCTDs such as joint hypermobility, skin extensibility, and easy bruising/bleeding, a specific diagnosis likely requires the aid of genomic technologies and NGS. Commonly, a particular patient presenting to their primary care physician requires a referral to a clinical geneticist for further evaluation, appropriate genetic testing and explanation of findings impacting care and risk for other family members. Though genetic testing is becoming more common and readily available, it is important for physicians to have knowledge regarding the most prevalent signs/symptoms in HCTDs to facilitate rapid identification of patients for referral and genetic evaluation by a clinical geneticist using disease specific genetic testing approaches.

Furthermore, it is possible that phenotypic heterogeneity within syndromes with well-described pathogenic variants is influenced by variants of unknown significance in other genes. For example, a study that examined a large group of patients with genotypically confirmed Marfan syndrome (i.e. identified pathogenic variants in the *FBN1* gene) found strong phenotypic correlations within a given organ system (skeletal, cardiovascular, ocular) but weak correlations between features belonging to different systems [16]. This suggests that pathogenic variants in the *FBN1* gene are not the only genetic factors contributing to expression of symptoms in Marfan syndrome [17]. Ultimately, to better counsel patients with HCTDs it is important to consider the possibility that variants with currently unknown significance are potentially pathogenic and require additional evaluation to fully assess the likelihood of damaging the protein product [18].

This study aimed to characterize the clinical and genetic findings of 100 consecutive, unrelated patients referred to the Genetics Clinic at the University of Kansas Medical Center. All patients had features of a connective tissue disorder, were examined by the senior author (MGB), an ABMG board-certified clinical geneticist, and underwent genetic testing with NGS connective tissue disorder gene panels. This study summarizes clinical and genetic findings to yield a better understanding of the type and frequency of presenting features for HCTDs. In addition, we characterize genotype–phenotype relationships and interactions of clinical features with molecular genetics, including providing evidence for potentially important variants that are not currently considered pathogenic or likely pathogenic but should be further studied for possible pathogenicity. To determine if unique classes of symptoms were present among patients, a latent class analysis was used.

## Methods

### Participant selection, study setting and design

Participants included patients presenting over a 3.5-year period (2016–2020) to the University of Kansas Medical Center Genetics Clinic with features of HCTDs (e.g., an elevated Beighton flexibility score, joint instability, subluxations and pain, aneurysms, scoliosis, skin fragility and easy bruising with or without a positive family history) [14]. Each participant was evaluated by a board certified clinical geneticist (MGB) in the Genetics Clinic including: (1) a standard clinical evaluation for chief complaint, history of present illness, past medical history, allergies and medications, 13-item review of systems, three generation family history, height, weight, blood pressure and heart rate, detailed physical examination of all organ systems with Beighton hypermobile score recorded along with a development of plan and (2) assessment with next generation sequencing and deletion/duplication status of a comprehensive connective tissue disorder gene panel consisting of 74 genes based on the clinical context of the patient, medical and family histories and clinical presentation. Sequenced genes included the approved connective tissue panel from Fulgent (https://www.fulgentgenetics.com/Connective-Tissue) as well as additional genes selected based on current evidence in the field and the expertise of the senior author (Additional file 1: Table S1). Each participant agreed and signed the genetic testing order form and subsequent usage of de-identified clinical information required for clinical care, surveillance and treatment. Patients were enrolled regardless of age, gender or ethnicity to help reduce potential bias associated with these characteristics. Participants were only required to be unrelated and have available results from a custom connective tissue disorder panel consisting of 74 genes performed at Fulgent Genetics (Temple City, CA), a CLIA-approved and accredited commercial laboratory following approved testing guidelines required for certification. Ultimately, data from 100 consecutive, unrelated patients presenting for genetic services were included in a cross-sectional study evaluating influences of genetic variants on symptom expression.

### Data collection protocol

Clinical information was collected during the clinic visit along with buccal cells sent to the laboratory for DNA isolation and NGS connective tissue disorder testing. Data were de-identified, entered and stored with physical copies of the visits under lock and key with access restricted to approved study personnel only.

### Clinical and genetic measures

- Patient age, gender, chief complaint, review of systems, medical and family histories of HCTDs, inheritance patterns and physical examination. As a part of standard procedure during the detailed clinical evaluation, patients were also asked about symptoms relating to autonomic dysfunction, tachycardia, history of gastrointestinal issues (e.g., diarrhea, constipation, celiac disease, food allergies), joint pain and hypermobility, etc. These responses were recorded and utilized for assessment and plan of care for delivery of patient services.
- Phenotypic features recorded were based on those known to be associated with clinically suspected heritable connective tissue disorders, clinical context and included Beighton hyperflexibility scores greater than or equal to 5 out of 9 and 33 identified clinical symptoms stratified in seven categories or systems as follows: skeletal, connective tissue, skin, eye, nervous, gastrointestinal and cardiovascular with list of features noted within each of the seven clinical categories seen in Table 1.
- Results from NGS data analyses, or by Sanger sequencing to ensure 100% coverage of coding sequences, were manually extracted from Fulgent Genetics (fulgentgenetics.com) genetic testing reports and included gene name, inheritance modality, deletions/duplications confirmed by an orthogonal method (qPCR or MLPA), variant type (missense, nonsense, frameshift, indel) and position based on Hg19 build, amino acid substitution (if applicable), zygosity, and pathogenicity classification (pathogenic, likely pathogenic, unknown clinical significance) following American College of Medical Genetics established guidelines. As this study aimed to describe variants of unknown clinical significance (VUS) that were potentially deleterious leading to pathogenicity, VUS were further interrogated and considered potentially pathogenic, if meeting at least three out of four criteria provided by Fulgent Genetics in genetic testing reports that were selected a priori based on the expertise of the practicing clinical geneticist and the clinical context of the patient history and presentation included: (1) maximum allele frequency ≤ 0.03% in the Broad Institute gnomAD database, (2) missense variant Grantham distance > 100 reflecting the evolutionary distance between the referent amino acid and the substituted amino acid (distances range from 5 to 250), (3) amino acid evolutionary conservation ≥ 90% across all mammals (including primates), and (4) in silico predictive computational scores with ≥ 50% deleterious changes identified in up to 10 programs used by the genetic testing laboratory over the 3.5-year period (2016–2020) during which time DNA was sent for testing. As such, not all variants identified were evaluated using all 10 programs as some reflected more recent predictors that may not have yet been available or were unable to provide a prediction due to limited information in the literature at the time of genetic testing. These ten programs were AGVGD, FATHMMMKL, LRT, SIFT, MUTATION ASSESSOR, MUTATION TASTER, FATHMM, METALR, METASVM and PROVEAN. Details on potentially pathogenic variants are provided in Additional file 2: Table S2. The remaining VUSs were classified as ‘other’.

**Table 1:** Clinical signs and symptoms of 100 consecutive patients presenting for genetic services with features of a connective tissue disorder

System	Clinical feature	Patients presenting with clinical features (%)
Skeletal	Limb Asymmetry	30
	Scoliosis	23
	Pes Planus	14
	Spine Anomaly	9
	Chest Deformity	7
	Kyphosis	2
Connective Tissue	Joint Hypermobility	79
	Joint Subluxation	51
	Dental Defects	15
	Temporomandibular Joint Dysfunction	14
	Repeated Ligament & Cartilage Damage	14
Skin	Hyperextensible/Loose Skin	69
	Easy Bruising/Bleeding	59
	Poor Wound Healing/Striae	37
Eye	Myopia	29
	Cataracts	4
	Corneal Defect	2
	Glaucoma	1
Nervous	Migraines	28
	Chronic Fatigue	19
	Neuropathy	14
	Tinnitus	13
Gastrointestinal	Irritable Bowel Syndrome	17
	Food Intolerance	16
	Chronic Constipation/Diarrhea	12
	Celiac	9
Cardiovascular	Cardiac Valve/Septal Defect	17
	Peripheral Vascular Disease	9
	Postural Orthostatic Tachycardia Syndrome	7
	Aneurysm	3

### Statistical analyses

The male-to-female ratio and averages/ranges of ages and Beighton scores were calculated for the dataset. Frequencies of specific symptoms categorized as present or absent and overall prevalence within a system were recorded. Presence of pathogenic, likely pathogenic, or potentially pathogenic VUSs were evaluated in relation to clinical features and the average number of these types of variants identified per patient was calculated.

Considering patients presented to the genetics clinic due to suspicion of any HCTD and many genetic testing results were inconclusive, to better understand the relationships among symptoms and determine if we could identify more homogenous groups based on symptom profiles that may reflect distinct HCTDs (e.g., EDS vs Marfan syndrome), we performed latent class analysis (LCA). We used the polytomous variable Latent Class Analysis (poLCA) package in R v 1.4.1 [19]. LCA assumes variable independence; to meet assumptions of subsequent modeling we first performed chi-square tests to determine if presence of any symptoms defined within the above mentioned seven organ systems were not independent. To ensure a stringent criterion for independence of symptoms included in LCA, the threshold for significant evidence of dependence between symptom categories was set at an uncorrected *p* < 0.10. As the *p* value represents the probability that a relationship between any two tested symptoms would occur by random chance, in this case that threshold was set at less than a 90% chance. For systems where symptoms were evidenced to be associated, we additionally evaluated the associations in logistic regression models while adjusting for age, gender and variant class—where variant class was categorized based on having at least one variant of interest (i.e., pathogenic, likely pathogenic or potentially pathogenic) versus having other VUS not evidenced to be deleterious or no variants identified. False discovery rate was also controlled for using the Benjamini–Hochberg procedure and adjusted *p* values are reported to allow for comprehensive presentation of the results of these initial exploratory analyses.

Four LCA models that included independent symptom variables were fit assessing the possibility of one to seven latent classes, with and without estimates of covariate effects (i.e., age and gender) on underlying and measured variables. ‘Model 1’ included symptoms in the skeletal, connective tissue, nervous, gastrointestinal, and cardiovascular systems. ‘Model 2’ included these symptoms plus age and gender as covariates. ‘Model 3’ included symptoms in the skeletal, skin, eye, nervous, gastrointestinal, and cardiovascular systems. ‘Model 4’ included these symptoms plus covariates. Model fit and optimal number of latent classes were determined based on the best estimates of the Akaike information criterion (AIC), Bayesian information criterion (BIC), Pearson’s chi-square goodness of fit (Chisq), and likelihood ratio chi-square (Gsq) statistics that were calculated following 10 repetitions of each analysis. Considering all statistics calculated reflected different assumptions with different properties, the optimal LCA solution was determined to be the model where all four statistics were collectively minimized, while also maintaining reduced numbers of classes [19]. Notably, some class solutions and models reflected the lowest value calculated for one statistic but reflected the maximum values calculated for others. Ultimately, the final model and number of classes where all four statistics were collectively minimized also reflected the lowest value calculated for the AIC, which also maintained some of the lowest values that were calculated for BIC, Chisq and Gsq.

Fisher’s exact tests were used to compare the proportion of patients with symptoms in organ systems of interest across the final solution of latent classes. Considering the goals of these tests were to evaluate evidence for symptoms differences among patients assigned to latent classes (e.g., Class 1 vs Class 2, Class 2 vs Class 3, Class 1 vs Class 3), significance was based on a Benjamini–Hochberg adjusted *p* value < 0.05. Age was compared across all classes using one-way analysis of variance and between classes with t-tests. To determine if the latent symptom classes reflected genetic differences, logistic regression was used—while adjusting for age and gender—to test if having variants in any of the evaluated genes, or if specifically having genetic variants categorized as pathogenic, likely pathogenic or potentially pathogenic in any evaluated genes were associated with latent symptom classes.

## Results

### Demographics

Over an approximate 3.5-year period (2016–2020), there were 111 patients referred to the clinic for potential HCTDs presenting with concerns about a connective tissue disorder involving any or most of the seven clinical categories for connective tissue disorders as described in Table 1 with a Beighton hyperflexible score greater than 5 supporting a diagnosis of a connective tissue disorder in the majority of patients. Of these, four patients were excluded due to missing clinical information or genetic results and seven were excluded due to familial relation to another study participant, with only data from the initial presenting family member included. The final analysis dataset consisted of 100 unrelated participants. There were 80 females and 20 males with an average age (± SD) of 33 ± 14 years old. The range of ages within this cohort was 7 to 68 years old. The most common reason for presentation was a suspected unspecified connective tissue disorder (N = 71), followed by EDS (N = 18), joint hyperflexibility (N = 7), Marfan syndrome (N = 3), and Chiari malformation (N = 1).

### Clinical signs and symptoms

The average Beighton hyperflexibility score (± SD) was 5.9 ± 1.9 in 88 patients. Twelve patients were unable to be assessed due to joint pain, damage or surgery. A score of 9 out of 9 was observed in 9.1% of patients and a minimum score of zero observed in 1.1%. Within the skeletal system, the most common signs/symptoms were limb asymmetry—referring to different leg lengths and may be further impacted by flat feet, hip subluxation/dislocation and ankle instability (30%), followed by scoliosis (23%) and pes planus (14%). The most common connective tissue features within the patient cohort were joint hypermobility (79%), joint subluxation (51%), and dental defects (15%). Findings related to skin were quite common within the patient population: stretchable skin (69%), easy bruising/bleeding (59%), and poor wound healing/striae (37%). Ocular symptoms were observed with myopia at 29%. The two most common nervous system-related symptoms were migraines (28%) and chronic fatigue (19%). Gastrointestinal-associated problems included irritable bowel syndrome (17%) and food intolerance (16%). Cardiovascular findings included cardiac valve/septal defects reported in 17% of the patients. None of the patients reported a history of retinal detachment, spontaneous pneumothorax, or myocardial infarctions. The complete list of 30 clinical symptoms identified in the patient cohort categorized or grouped across seven organ systems can be seen in Table 1. The 30 clinical features categorized into the seven systems having the largest percentage among our patients presenting for services were joint hypermobility at 79% followed by loose skin at 69% and easy bruising at 59%. Those clinical features with the lowest occurrence were eye defects at 2%, kyphosis at 2% and glaucoma at 1%. The largest average Beighton score was seen in the connective tissue category at 6.3 and followed by 6.2 in the skeletal category.

### Genotypic-phenotypic features in patients presenting with connective tissue disorder

**Fig. 1:**
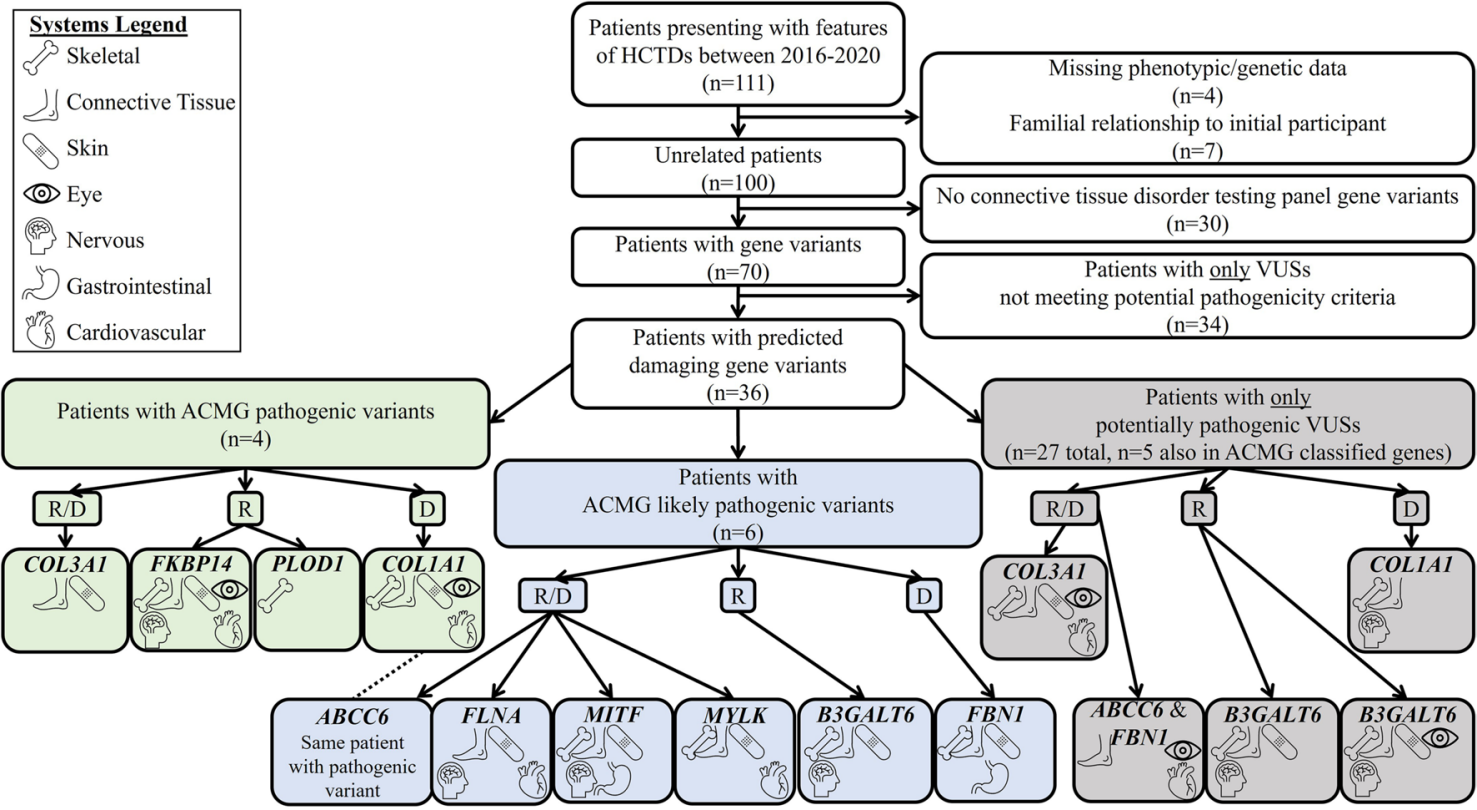
Genetic and phenotypic profiles of patients presenting for heritable connective tissue disorders (HCTDs). Flow chart with details for patients who presented to the genetics clinic over a 3.5 year period. Presence of symptoms observed across seven biological systems and next-generation sequencing results for 74 genes included on commercially available connective tissue disorder testing panels were evaluated for 100 unrelated patients. Variants reported as unknown clinical significance (VUS) according to ACMG classifications were further evaluated for potential pathogenicity based on allele frequency, biological conservation, Grantham distance and damaging in silico predictions. Shown are system symptom comparisons for patients with pathogenic (in green) or likely pathogenic (in blue) variants in the same genes as those with potentially pathogenic VUSs (in gray). Reported inheritance patterns are also noted; R = autosomal or X-linked recessive, D = autosomal or X-linked dominant, R/D indicates both autosomal or X-linked recessive and dominant have been reported

In total, there were 117 unique variants identified in 45 of the 74 genes included on commercially available connective tissue disorder testing panels (see Additional file 1: Table S1) encompassing seven clinical connective tissue-related systems (skeletal, connective tissue, skin, eye, nervous, gastrointestinal and cardiovascular). Of these, there were four ACMG-classified pathogenic and six likely pathogenic variants. In addition, 35 VUS in 31 genes were identified that met criteria for being potentially pathogenic based on the clinical context of the patient and gene variant found while 72 other VUSs met less than three prediction criteria and clinical context of each patient presenting for genetic services used to consider potential pathogenicity. Of the 35 potentially pathogenic gene variants which were seen in 30 unique patients, 12 of these variants were seen in genes with autosomal recessive inheritance and all variants included within the table were heterozygous (Additional file 2: Table S2). There were nine patients with potentially pathogenic VUS in genes reported to follow a recessive inheritance pattern who had no other variants of interest and expressed a range of symptoms. Notably, some genes were reported to have variants inherited in both recessive and dominant patterns (Fig. 1, Additional file 2: Table S2) and VUS are often not well-characterized making it difficult to define the influence of heterozygous potentially pathogenic VUS in genes previously reported to show recessive inheritance. There were also several patients identified who had more than one variant classified as either pathogenic, likely pathogenic or potentially pathogenic in distinct genes. On average, patients had 1.5 ± 0.9 variants (range 0–7) when considering all possible classifications; when focusing on pathogenic, likely pathogenic or potentially pathogenic VUSs there were 1.3 ± 0.6 variants (range 0–4) identified per patient. There were 30 patients where no variants were identified in suspected connective tissue disorder genes. A summary of the clinical findings grouped into seven categories in relationship to identified genetic variants is provided in Fig. 1 and Additional file 3: Table S3.

### Symptoms in patients with pathogenic, likely pathogenic and potentially pathogenic variants

To better understand if any potentially pathogenic variants reported to be of unknown significance according to ACMG classifications may be clinically relevant, symptom profiles for patients with an ACMG-classified pathogenic or likely pathogenic variant were compared with those of patients who had a potentially pathogenic variant identified in the same gene. Specifically, a pathogenic variant in *COL1A1* (p.Gly266Arg) was heterozygous in one patient (ID #54) with symptoms related to skeletal, connective tissue, skin, eye and cardiovascular issues. One other patient (ID #109) had a VUS identified in *COL1A1* that was potentially pathogenic (p.Arg796Cys). This patient also had skeletal and connective tissue symptoms, but no skin, eye or cardiovascular symptoms; however, they did have nervous system symptoms which were absent in the patient with the pathogenic variant in *COL1A1*. Notably, patient #54 with the pathogenic *COL1A1* variant also had a likely pathogenic intronic *ABCC6* variant (c.3883-6G > A). This likely pathogenic *ABCC6* variant is reported to exhibit autosomal recessive inheritance making it unlikely this variant modifies the effects of the pathogenic variant. Regardless, there was one patient (ID #7) with a potentially pathogenic VUS in *ABCC6* (p.Ala343Thr) exhibiting connective tissue, eye and cardiovascular symptoms; however, this patient also had a potentially pathogenic VUS in a different gene, *FBN1* (p.Arg1428His).

Other pathogenic variants of interest included a variant (c.1662 + 1G > A) near the *COL3A1* gene. This patient (ID #4) had symptoms related to connective tissue and skin. One other patient (ID #70) with a potentially pathogenic VUS identified in *COL3A1* (p.Glu745Lys) had skeletal, connective tissue, skin, eye and cardiovascular symptoms.

Finally, there were two patients with pathogenic variants identified in genes reported to only have a recessive inheritance pattern. One patient (ID #99) was heterozygous for a pathogenic deletion of the entire *FKBP14* gene with skeletal, connective tissue, skin, eye, nervous system, and cardiovascular symptoms. Another patient (ID #33) was heterozygous for a pathogenic duplication in *PLOD1* (p.Glu326Lys585dup) and only exhibited skeletal symptoms. No other patients had *FKBP14* or *PLOD1* variants.

Likely pathogenic variants were also identified in six patients. One patient (ID #80) had a likely pathogenic, heterozygous *B3GALT6* variant (p.Met1Val) and skeletal, connective tissue, skin and nervous system symptoms. In total, there were three patients with variants of potential interest in *B3GALT6*. One additional patient (ID #91) had four distinct potentially pathogenic VUSs identified including one in *B3GALT6* (p.Trp198Leu), as well as potentially pathogenic variants in *COL1A2* (p.Leu1019Phe), *FBN2* (p.Ile998Asn) and *TGFBR2* (p.Gly2Cys). The other patient (ID #104) had a potentially pathogenic *B3GALT6* VUS (p.Ala114Glu) and no other variants of interest. The common features among all patients with *B3GALT6* variants included skeletal, connective tissue, skin and nervous system symptoms although all patients were heterozygous. Variants in *B3GALT6* have only previously been reported to be inherited in a recessive pattern. As such, it remains unclear if heterozygous variants in this gene influence symptom expression.

In addition, a likely pathogenic X-linked dominant variant in *FLNA* (p.Ile137Asn) was heterozygous in one female patient (ID #73) with connective tissue, skin, nervous system and cardiovascular symptoms. A likely pathogenic variant was identified in *MITF* (p.Glu318Lys) in a patient (ID #77) with skeletal, connective tissue, skin, nervous system and gastrointestinal symptoms. A likely pathogenic variant was identified in *MYLK* (p.Thr165Profs*72) in one patient (ID #108) with skeletal, connective tissue, skin and cardiovascular issues. Symptom comparisons between patients with likely pathogenic and potentially pathogenic variants could not be conducted as no other patients had variants of interest identified in *FLNA*, *MITF*, or *MYLK*.

With regard to the specific set of 19 genes where variation is reported to cause EDS [10], there were 14 patients with variants of interest in eight known EDS genes. These included the above listed genes, *B3GALT6*, *COL1A1*, *COL1A2*, *COL3A1*, *FKBP14*, and *PLOD1*. In addition, potentially pathogenic VUS were identified in *COL12A1* (reported to be inherited in an autosomal dominant pattern) and *ZNF469* (currently reported recessive); however, no ACMG-classified pathogenic or likely pathogenic variants were identified in either *COL12A1* or *ZNF469* in this patient cohort and symptom profiles could not be compared.

### Relationships among symptoms in the patient cohort

As most patients had either no variants, or only variants of unknown clinical significance identified in connective tissue panel genes, it was difficult to confirm an HCTD using genetic testing results. To better understand the phenotypic heterogeneity in patients presenting for suspicion of these disorders, symptom presentation was comprehensively characterized across the entire dataset. To ensure LCA assumptions were met, exploratory tests of independence among symptoms in the organ systems of interest in the full dataset were conducted and are provided in Table 2. Potential relationships were observed between the presence of connective tissue symptoms and skin (OR 2.18, *p* = 0.02) and eye symptoms (OR 1.89, *p* = 0.06); no other relationships were identified based on an unadjusted *p* < 0.10 (Table 2). Male gender was evidenced to possibly influence the relationship between the presence of connective tissue and eye symptoms (Table 3).

**Table 2:** **Relationships among symptoms across seven organ systems in HCTD patients**. Shown are results of chi-square (χ2) tests comparing symptoms among seven organ systems in patients with heritable connective tissue disorders (HCTDs). Systems evidenced to be independent, based on unadjusted p ≥ 0.10, were subsequently included in LCA models. Specific clinical features and percentages reflecting the overall system with symptoms present can be found in Table 1. Included are Benjamini–Hochberg adjusted *p* values. Expected counts were rounded to the nearest whole number. Abbreviations: obs = observed, exp = expected, OR = odds ratio, CI = confidence interval

Symptoms in both systems		χ^2^	OR (95%CI)	*p* value	*p* value_adjusted_
*Skeletal*					
Connective Tissue	obs = 47; exp = 50	1.58	1.52 (0.79, 2.91)	0.21	0.58
Skin	obs = 51; exp = 49	0.74	1.33 (0.69, 2.55)	0.39	0.70
Eye	obs = 21; exp = 20	0.05	1.32 (0.69, 2.53)	0.82	1.00
Nervous	obs = 30; exp = 29	0.16	1.62 (0.84, 3.12)	0.69	0.97
Gastrointestinal	obs = 22; exp = 23	0.02	1.51 (0.79, 2.89)	0.90	1.00
Cardiovascular	obs = 19; exp = 16	1.31	1.40 (0.73, 2.68)	0.25	0.58
*Connective tissue*					
Skin	obs = 78; exp = 75	5.27	2.18 (1.12, 4.24)	0.02	0.42
Eye	obs = 34; exp = 30	3.62	1.89 (0.98, 3.66)	0.06	0.58
Nervous	obs = 46; exp = 44	1.41	1.45 (0.77, 2.85)	0.23	0.58
Gastrointestinal	obs = 38; exp = 35	2.69	1.73 (0.90, 3.33)	0.10	0.86
Cardiovascular	obs = 23; exp = 24	0.32	1.21 (0.63, 2.31)	0.57	0.86
*Skin*					
Eye	obs = 32; exp = 30	0.72	1.32 (0.69, 2.53)	0.40	0.70
Nervous	obs = 46; exp = 43	2.08	1.62 (0.84, 3.11)	0.15	0.58
Gastrointestinal	obs = 37; exp = 34	1.53	1.51 (0.79, 2.89)	0.22	0.58
Cardiovascular	obs = 22; exp = 24	1.03	1.40 (0.73, 2.68)	0.31	0.65
*Eye*					
Nervous	obs = 21; exp = 18	1.58	1.52 (0.79, 2.92)	0.21	0.58
Gastrointestinal	obs = 14; exp = 14	0.00	NA	1.00	1.00
Cardiovascular	obs = 10; exp = 10	2.70 × 10^–31^	1.00 (0.52, 1.91)	1.00	1.00
*Nervous System*					
Gastrointestinal	obs = 22; exp = 20	0.38	1.22 (0.64, 2.34)	0.54	0.86
Cardiovascular	obs = 13; exp = 14	0.05	1.08 (0.56, 2.06)	0.82	1.00
*Gastrointestinal*					
Cardiovascular	obs = 11; exp = 11	2.91 × 10^–31^	1.00 (0.52, 1.91)	1.00	1.00

**Table 3:** **Effects of covariates on relationships between associated symptoms**. Shown are results testing logistic regression models adjusting for age, gender and presence of pathogenic, likely pathogenic or potentially pathogenic variants. Abbreviations: OR = odds ratio, CI = confidence interval

Connective tissue symptom	OR (95%CI)	*p* value
Skin Symptom	2.98 (1.85, 4.11)	0.03
Age	− 0.06 (− 1.12, 1.00)	0.27
Male Gender	− 1.00 (− 2.07, 0.06)	0.58
Variant of Interest	0.51 (− 0.55, 1.57)	0.78
**Eye Symptom**	**5.79 (4.46, 7.12)**	**0.03**
Age	− 3.41 (− 4.56, − 2.25)	0.11
Male Gender	− 4.09 (− 5.29, − 2.89)	0.07
Variant of Interest	0.04 (− 1.02, 1.09)	0.98

**Fig. 2:**
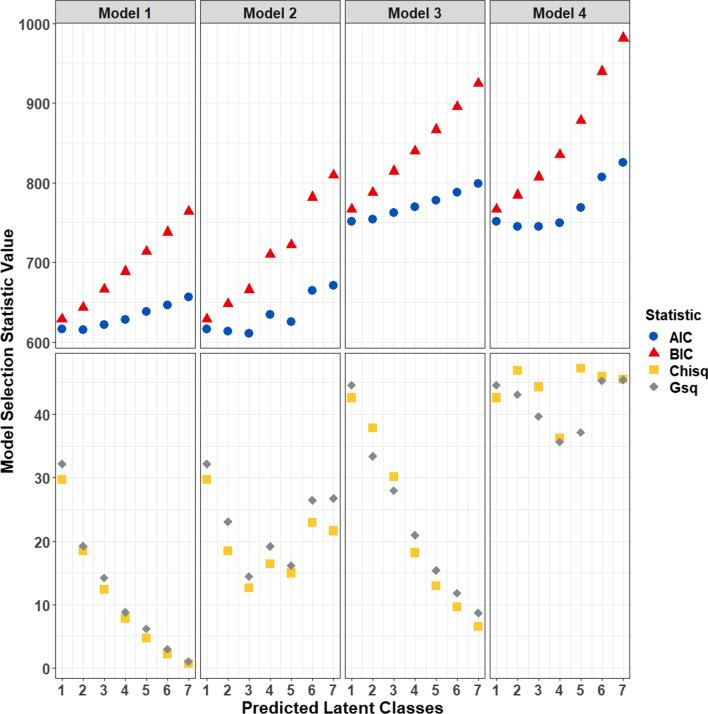
Detection of unique classes of symptom profiles among patients. Shown are statistics on the y-axis calculated for latent class analysis of the possibility of distinct patient groups based on four models of symptom and demographic profiles. For each model, the number of evaluated groups are on the x-axis and ranged from the undivided dataset (1 group) to seven groups. Models 1 and 2 evaluated patient classes based on symptoms in skeletal, connective tissue, nervous, gastrointestinal, and cardiovascular systems; Model 2 included age and gender. Models 3 and 4 evaluated skeletal, skin, eye, nervous, gastrointestinal, and cardiovascular system symptoms with age and gender in Model 4. The y-axis was split to visualize statistics measured on different scales. Akaike (AIC; blue circles) and Bayesian information criterion (BIC; red triangles) values ranged from 611 to 960. Pearson’s chi-square goodness of fit (Chisq; yellow squares) and likelihood ratio chi-square (Gsq; gray diamonds) values ranged from 2 to 48. The optimal result was achieved using Model 2 to group patients into three classes which reflected the lowest AIC value and maintained smaller values for other statistics

The best performing latent class analysis results were identified when dividing the entire dataset of HCTD patients into three distinct groups based on age, gender and presence or absence of symptoms in the skeletal, connective tissue, nervous, gastrointestinal, and cardiovascular systems (i.e., ‘Model 2’; Fig. 2). Observations of symptoms in all systems except for skin and eyes, as well as ages and gender were different among patients assigned to the three distinct classes, based on an adjusted *p* value < 0.05 (Table 4). Results of direct comparisons of symptoms and demographic characteristics between each of the distinct patient groups are provided in Table 5. When compared to the two other distinct groups of patients, there were 16 patients assigned to the first latent class, ‘Class 1’, who were younger (average age = 16 ± 8) males and females with fewer symptoms related to skeletal system disturbances. A second distinct class of 17 patients, ‘Class 2’, were older (average age = 36 ± 16) with more skeletal system symptoms when compared to Class 1 patients. Class 2 contained an almost equal distribution of males and female (female gender = 47%). In addition, individuals in Class 2 had fewer connective tissue symptoms than any other class of patients and no nervous system or gastrointestinal symptoms. A third class, ‘Class 3’, contained 67 mostly female patients (female gender = 90%) with comparable age (average age = 36 ± 12) to Class 2 patients. These patients had the most nervous system symptoms. Individuals in Class 3 also had more connective tissue and gastrointestinal symptoms when compared to patients in Class 2.

**Table 4:** **Differences in symptoms, ages, and gender of HCTD patients by latent class**. Reported are Fisher’s exact test results for differences in the number of patients with symptoms observed in evaluated organ systems among all of the latent classes (see Fig. 2 for details on latent class analysis results). The proportion of patients assigned to each class with the symptom, as well as comparisons of ages (along with means ± standard deviations and ranges) are provided. Specific clinical features and percentages reflecting the overall system with symptoms present can be found in Table *1*. Unadjusted and Benjamini–Hochberg adjusted *p* values are provided

Organ System	Class 1 (n = 16)	Class 2 (n = 17)	Class 3 (n = 67)	*p* value	*p* value_adjusted_
Skeletal	0.13	0.82	0.61	9.07 × 10^–5^	2.04 × 10^–4^
Connective Tissue	1.00	0.47	0.94	1.29 × 10^–5^	3.89 × 10^–5^
Skin	0.81	0.71	0.91	0.05	0.06
Eye	0.38	0.29	0.36	0.90	0.90
Nervous	0.06	0.00	0.73	3.04 × 10^–12^	2.73 × 10^–11^
Gastrointestinal	0.56	0.00	0.46	1.92 × 10^–4^	3.46 × 10^–4^
Cardiovascular	0.00	0.53	0.28	1.46 × 10^–3^	1.88 × 10^–3^
Gender Female	0.75	0.47	0.90	5.00 × 10^–4^	7.49 × 10^–4^
Age	16 ± 8 (3–30)	36 ± 16 (14–67)	36 ± 12 (15–68)	4.34 × 10^–7^	1.95 × 10^–6^

**Table 5:** **Direct comparisons of symptoms and demographics between latent class**. ﻿Reported are Fisher’s exact test results for differences in the number of patients with symptoms observed in evaluated organ systems between each of the latent classes (see Fig. 2 for details on latent class analysis results). Unadjusted and Benjamini–Hochberg adjusted *p* values are provided

Organ System	Latent Class Comparison	*p* value	*p* value_adjusted_
Skeletal	Class 1 versus Class 2	8.60 × 10^–5^	4.13 × 10^–4^
	Class 1 versus Class 3	5.62 × 10^–4^	1.35 × 10^–3^
	Class 2 versus Class 3	0.15	0.22
Connective Tissue	Class 1 versus Class 2	9.27 × 10^–4^	1.85 × 10^–3^
	Class 1 versus Class 3	1.00	1.00
	Class 2 versus Class 3	3.13 × 10^–5^	1.88 × 10^–4^
Skin	Class 1 versus Class 2	0.69	0.75
	Class 1 versus Class 3	0.37	0.46
	Class 2 versus Class 3	0.04	0.07
Nervous	Class 1 versus Class 2	0.49	0.58
	Class 1 versus Class 3	1.03 × 10^–6^	8.20 × 10^–6^
	Class 2 versus Class 3	1.78 × 10^–8^	2.13 × 10^–7^
Gastrointestinal	Class 1 versus Class 2	2.97 × 10^–4^	8.90 × 10^–4^
	Class 1 versus Class 3	0.58	0.66
	Class 2 versus Class 3	1.52 × 10^–4^	5.23 × 10^–4^
Cardiovascular	Class 1 versus Class 2	9.27 × 10^–4^	1.85 × 10^–3^
	Class 1 versus Class 3	0.02	0.03
	Class 2 versus Class 3	0.08	0.13
Gender	Class 1 versus Class 2	0.16	0.22
	Class 1 versus Class 3	0.21	0.28
	Class 2 versus Class 3	3.63 × 10^–4^	9.69 × 10^–4^
Age	Class 1 versus Class 2	1.35 × 10^–4^	5.23 × 10^–4^
	Class 1 versus Class 3	9.22 × 10^–8^	2.21 × 10^–8^
	Class 2 versus Class 3	0.97	1.00

There was no evidence that having any type of variant in any of the evaluated genes was associated with assignment to a latent symptom class (β = − 0.10, *p* = 0.78). Furthermore, having a variant classified as pathogenic, likely pathogenic, or potentially pathogenic VUS in any evaluated gene was not associated with the latent classes (β = 0.32, *p* = 0.23).

## Discussion

This study is one of the first to use latent class analysis to comprehensively characterize connective tissue disorder symptom profiles, and to relate this to genetic testing results by reporting genetic variation identified in this large dataset of individuals with HCTDs. To provide insight into the types of variants identified in genes involved with connective tissue disorders, results from NGS of patients referred for evaluation at an academic medical center genetics clinic were manually extracted from genetic testing reports and analyzed. Genetic testing results from 100 patients identified only four ACMG-classified pathogenic and six likely pathogenic variants. The majority of the variants identified in the known heritable connective tissue disorder genes included on genetic testing panels were of therefore unknown significance (VUS). This indicates the need to identify additional genes of interest for testing and that research should focus on better functional characterization of VUS to improve future genetic testing and interpretation of results. Notably, there were 35 VUSs in 31 genes that upon further evaluation were potentially pathogenic in approximately half (56%) of the genes currently included on these panels. Symptom presentation appeared similar when comparing data from individuals with pathogenic/likely pathogenic variants to those with potentially pathogenic VUS. Given the limitations of in silico predictors that were used to define potentially pathogenic variants, additional follow-up studies are necessary to confirm an effect on the protein product. Regardless, this suggests that the prediction criteria used to further delineate VUS to detect those that were likely damaging to protein products may be useful to helping understand how to better treat patients for whom a pathogenic or likely pathogenic variant is not identified.

Chi-square tests were used to evaluate relationships among symptoms in seven organ systems. Although results should be considered in the context of these exploratory analyses and did not survive correction for multiple testing, given the presence of a connective tissue symptom (e.g., joint hypermobility, joint subluxation, dental defects, temporomandibular joint dysfunction, repeated ligament & cartilage damage), the odds of having a skin symptom (e.g., hyperextensible/loose skin, easy bruising/bleeding, poor wound healing/striae) were 2.18 times greater than the odds in the absence of connective tissue symptoms. Similarly, the odds of having an eye symptom (e.g., myopia, cataracts, corneal defect, glaucoma) were 1.89 times greater given connective tissue symptoms. Male gender was near significant (*p* = 0.07) for reducing the odds of having comorbid eye and connective tissue symptoms. Furthermore, relationships between connective tissue and gastrointestinal symptoms were near significance (*p* = 0.10) and the next largest observed effect (OR 1.73).

Latent class analysis was used to look for underlying subgroups of patients with similar symptom profiles. There were three distinct groups of patients with variable likelihood of skeletal, connective tissue, nervous and gastrointestinal symptoms. One class of younger patients was identified with no evidence of cardiovascular problems, which was not surprising given their ages. However, it was notable that these patients had less evidence of skeletal symptoms and very few nervous system symptoms. A second class of patients was identified that could be distinguished by having no evidence of nervous system or gastrointestinal issues. Finally, the largest subgroup of patients was those with the most organ systems impacted and could be distinguished based on more nervous system symptoms.

Four patients, out of the 100 included in the dataset (4%), were heterozygous for variants classified as pathogenic. One patient had a pathogenic *COL1A1* variant and exhibited symptoms related to skeletal system dysfunction. Pathogenic variants in *COL1A1* are inherited in an autosomal dominant pattern and are causal for osteogenesis imperfecta type IV [20]. In addition, a likely pathogenic variant in the *ABCC6* gene was heterozygous in the same patient; however, this variant is evidenced to be recessive [21], and is unlikely to be responsible for the clinical presentation. One patient had a pathogenic variant in *COL3A1* and connective tissue and skin symptoms. Pathogenic variants in *COL3A1* are inherited in an autosomal dominant pattern and are causal for vascular EDS type IV [22], indicating this variant was responsible for these issues. A patient with a pathogenic variant in *FKBP14* had connective tissue symptoms. As pathogenic variation in this gene are autosomal recessive causing kyphoscoliosis EDS type II [23], it is unclear if this patient’s symptoms can be attributed to the variant. No other variants of interest were identified in this patient suggesting the possibility of additional inheritance patterns involved in expression of symptoms when this gene is deleted. Finally, one patient had a pathogenic *PLOD1* variant and scoliosis but not connective tissue symptoms. This variant in *PLOD1* is autosomal recessive and causal for kyphoscoliosis EDS type I [24]. In addition, likely pathogenic variants were identified in *FBN1* (N = 1 patient), *FLNA* (N = 1 patient) and *MYLK* (N = 1 patient) causing classic genetic disorders such as Marfan syndrome (*FBN1*; autosomal dominant), Melnick-Needles syndrome (*FLNA*; X-linked dominant) and familial thoracic aortic aneurysm (*MYLK*; autosomal dominant); all patients had significant health concerns consistent with these disorders.

There were several variants identified that were currently classified as unknown significance and potentially pathogenic based on likelihood of being damaging to the gene product. These may be of particular interest for future evaluation and functional characterization. For example, similar to the patient with the likely pathogenic *FBN1* variant, the patient with the potentially pathogenic variant had features consistent with Marfan syndrome (e.g., joint subluxations, double-jointedness, tall stature, long arms and thin body habitus) [16, 17]. One other individual with a *FBN1* variant that did not meet pathogenicity criteria exhibited some traits like Marfan syndrome but not the trademark tall stature. There were also two patients with variants in the *COL3A1* gene where known pathogenic variants are autosomal dominant and causal for Ehlers Danlos syndrome type IV and rare type III [25]. One patient had a pathogenic variant while the other had a potentially pathogenic VUS. Both patients had evidence of connective tissue and skin symptoms. Notably, the individual with the potentially pathogenic VUS had symptoms in more systems than the patient with the pathogenic variant. There were also three patients with differentially classified variants in *COL1A1*. Pathogenic variants in *COL1A1* are causal for Ehlers–Danlos syndrome (type I and VII), osteogenesis imperfecta (type I-IV), osteoporosis, and Caffey diseases (https://www.omim.org/entry/120150). All three patients had scoliosis; however, this was the only noted symptom for the patient with the VUS not meeting pathogenicity criteria. The two other patients had several skeletal and connective tissue issues.

### Strengths and limitations

These analyses offer insight into distinguishing characteristics among different patients with HCTD; however, particularly with regard to the identification of latent symptom classes, results should be considered preliminary and require replication in independent datasets to further confirm these findings. While the evaluated sample size is relatively small for LCA, we attempted to maximize performance by including high quality indicators that were confirmed via detailed clinical evaluations, as well as including covariate effects related to age and gender. This approach has been shown to help compensate for small sample size and be generally beneficial to improving performance based on evidence from Monte Carlo simulations that evaluated LCA for samples sizes ranging from 100 to 2000 [26]. Regardless, our results require replication in additional, larger datasets of patients with HCTDs. This is especially important considering the substantial genetic and phenotypic heterogeneity that was observed in this patient cohort which makes it difficult to robustly establish phenotype-genotype relationships in small samples. Although the genetic data included in this work reflects the most appropriate approach to genetic testing with regard to clinical applicability, a potential limitation of this study is that only variants in currently implicated connective tissue genes were evaluated. Notably, there were 30 patients where no evaluated gene was observed to have a variant. We also did not observe differences in the probability of having variants of any pathogenicity classification in the evaluated genes based on assignment to a distinct symptom class. This indicates that distinct symptom profiles may not relate to variation in genes that are currently implicated in expression of HCTDs. It is possible that there are yet unknown genes where variation influences risk for HCTDs and specific subtypes of HCTDs. Future work may expand genetic analysis to encompass additional genes to help determine if there are distinct genetic contributions to having an HCTD with a particular symptom profile.

## Conclusions

The analytical methods used reflect novel approaches for characterizing clinical symptoms and relationships in those patients presenting with features of HCTDs and should stimulate additional research into genotype–phenotype relationships. Clinical presentation and gene findings could have consequences for diagnosis, surveillance and counseling for the patient, guidance for health care providers, organ system surveillance and for evaluating at risk family members.

## Supplementary Information

**Additional file 1: Table S1**. Connective Tissue Disorder Genes Included on NGS Sequencing Panels.

**Additional file 2: Table S2**. Connective Tissue Disorder Gene Variants of Unknown Significance Meeting Potential Pathogenicity Criteria and Clinical Context.

**Additional file 3: Table S3**. Genotypic and Phenotypic Profiles of 100 Patients Presenting for Suspicion of Heritable Connective Tissue Disorders.

## Data Availability

Data are available from the authors upon reasonable request.

## Abbreviations

ABMG: American Board of Medical Genetics and Genomics
ACMG: American College of Medical Genetics and Genomics
AIC: Akaike information criterion
BIC: Bayesian information criterion
Chisq: Pearson’s chi-square goodness of fit
CI: Confidence interval
CLIA: Clinical Laboratory Improvement Amendments
EDS: Ehlers–Danlos syndrome
EXP: Expected
Gsq: Likelihood ratio chi-square
HCTDs: Heritable connective tissue disorders
LCA: Latent class analysis
MLPA: Multiple ligation probe amplification
NGS: Next-generation sequencing
OBS: Observed
OR: Odds ratio
qPCR: Quantitative polymerase chain reaction
SD: Standard deviation
VUS: Variants of unknown clinical significance
