# Supplementary material for: Clinical genetics evaluation and testing of connective tissue disorders: a cross-sectional study
Source: BMC Med Genomics. 2022 Aug 2;15:169. doi: 10.1186/s12920-022-01321-w (PMC9344629; doi:10.1186/s12920-022-01321-w)
Supplement: Supplementary file 1 — Additional file 1: Table S1. Connective Tissue Disorder Genes Included on NGS Sequencing Panels. [file 12920_2022_1321_MOESM1_ESM.docx]

| **Supplemental Table 1. Connective Tissue Disorder Genes Included on NGS Sequencing Panels.** | |
| --- | --- |
| **Gene Symbol** | **Gene Description** |
| *ABCC6* | ATP binding cassette subfamily C member 6 |
| *ACTA2* | actin alpha 2, smooth muscle |
| *ADAMTS10* | ADAM metallopeptidase with thrombospondin type 1 motif 10 |
| *ADAMTS2** | ADAM metallopeptidase with thrombospondin type 1 motif 2 |
| *ADAMTSL2* | ADAMTS like 2 |
| *AEBP1* | AE binding protein 1 |
| *ALDH18A1* | aldehyde dehydrogenase 18 family member A1 |
| *ATP6V0A2* | ATPase H+ transporting V0 subunit a2 |
| *ATP6V1E1* | ATPase H+ transporting V1 subunit E1 |
| *ATP7A* | ATPase copper transporting alpha |
| *B3GALT6** | beta-1,3-galactosyltransferase 6 |
| *B3GAT3* | beta-1,3-glucuronyltransferase 3 |
| *B4GALT7** | beta-1,4-galactosyltransferase 7 |
| *BGN* | biglycan |
| *C1R** | complement C1r |
| *C1S** | complement C1s |
| *CBS* | cystathionine beta-synthase |
| *CHST14** | carbohydrate sulfotransferase 14 |
| *COL11A1* | collagen type XI alpha 1 chain |
| *COL11A2* | collagen type XI alpha 2 chain |
| *COL12A1** | collagen type XII alpha 1 chain |
| *COL1A1** | collagen type I alpha 1 chain |
| *COL1A2** | collagen type I alpha 2 chain |
| *COL2A1* | collagen type II alpha 1 chain |
| *COL3A1** | collagen type III alpha 1 chain |
| *COL4A1* | collagen type IV alpha 1 chain |
| *COL5A1** | collagen type V alpha 1 chain |
| *COL5A2** | collagen type V alpha 2 chain |
| *COL9A1* | collagen type IX alpha 1 chain |
| *COL9A2* | collagen type IX alpha 2 chain |
| *CRTAP* | cartilage associated protein |
| *DSE** | dermatan sulfate epimerase |
| *EFEMP2* | EGF containing fibulin extracellular matrix protein 2 |
| *ELN* | elastin |
| *FBLN5* | fibulin 5 |
| *FBN1* | fibrillin 1 |
| *FBN2* | fibrillin 2 |
| *FKBP14** | FKBP prolyl isomerase 14 |
| *FLCN* | folliculin |
| *FLNA* | filamin A |
| *FOXE3* | forkhead box E3 |
| *GORAB* | golgin, RAB6 interacting |
| *LOX* | lysyl oxidase |
| *LTBP4* | latent transforming growth factor beta binding protein 4 |
| *MAT2A* | methionine adenosyltransferase 2A |
| *MED12* | mediator complex subunit 12 |
| *MFAPS* | NA |
| *MYH11* | myosin heavy chain 11 |
| *MYLK* | myosin light chain kinase |
| *NOTCH1* | notch receptor 1 |
| *NOTCH2* | notch receptor 2 |
| *P3H1* | prolyl 3-hydroxylase 1 |
| *PKD1* | polycystin 1, transient receptor potential channel interacting |
| *PLOD1** | procollagen-lysine,2-oxoglutarate 5-dioxygenase 1 |
| *PRDM5** | PR/SET domain 5 |
| *PRKG1* | protein kinase cGMP-dependent 1 |
| *PTDSS1* | phosphatidylserine synthase 1 |
| *PYCR1* | pyrroline-5-carboxylate reductase 1 |
| *RIN2* | Ras and Rab interactor 2 |
| *SGMS2* | sphingomyelin synthase 2 |
| *SKI* | SKI proto-oncogene |
| *SLC2A10* | solute carrier family 2 member 10 |
| *SLC39A13** | solute carrier family 39 member 13 |
| *SMAD2* | SMAD family member 2 |
| *SMAD3* | SMAD family member 3 |
| *SMAO4* | NA |
| *SMAD6* | SMAD family member 6 |
| *TAB2* | TGF-beta activated kinase 1 (MAP3K7) binding protein 2 |
| *TGFB2* | transforming growth factor beta 2 |
| *TGFB3* | transforming growth factor beta 3 |
| *TGFBR1* | transforming growth factor beta receptor 1 |
| *TGFBR2* | transforming growth factor beta receptor 2 |
| *TNXB** | tenascin XB |
| *ZNF469** | zinc finger protein 469 |
| Included are gene symbols and descriptions for all 74 genes sequenced by a CLIA-approved genetic testing laboratory (Fulgent Genetics) to detect variants that were ACMG classified as 'pathogenic', 'likely pathogenic', or 'unknown clinical significance' in the 100 patients presenting for suspicion of heritable connective tissue disorders. More information on the associated disorders and inheritance pattern of reported variants can be found in the Online Mendelian Inheritance in Man (<https://www.omim.org/>). *denotes Elhers- Danlos syndrome genes reported by Malfait et al. The 2017 international classification of the Ehlers–Danlos syndromes. 2017. Am J Med Genet Part C Semin Med Genet 175C:8–26, PMID: 28306229. | |
