# Supplementary material for: Clinical genetics evaluation and testing of connective tissue disorders: a cross-sectional study
Source: BMC Med Genomics. 2022 Aug 2;15:169. doi: 10.1186/s12920-022-01321-w (PMC9344629; doi:10.1186/s12920-022-01321-w)
Supplement: Supplementary file 2 — Additional file 2: Table S2. Connective Tissue Disorder Gene Variants of Unknown Significance Meeting Potential Pathogenicity Criteria and Clinical Context. [file 12920_2022_1321_MOESM2_ESM.docx]

| **Supplemental Table 2. Connective Tissue Disorder Gene Variants of Unknown Significance Meeting Potential Pathogenicity Criteria and Clinical Context.** | | | | | | | | | |
| --- | --- | --- | --- | --- | --- | --- | --- | --- | --- |
| **Patient ID** | **Gene Affected** | **Allele Substituted** | **Amino Acid Position & Change** | **Maximum Allele Frequency** | **Grantham's Distance** | **Mammalian Amino Acid Conservation** | ***In Silico* Deleterious Prediction** | **Inheritance Pattern** |  |
| 1 | *ZNF469* | c.8704G>T | p.Asp2902Tyr | **0.03%** | **160** | **100%** | **50%** | AR |  |
| 3 | *MED12** | c.5203C>T | p.Arg1735Cys | **0.00%** | **180** | **100%** | **50%** | XLD&XLR |  |
| 4 | *COL11A2* | c.1136G>A | p.Arg379Gln | **0.00%** | 42 | **98%** | **100%** | AD&AR |  |
| 5 | *ADAMTSL2* | c.1261G>A | p.Gly421Ser | **0.00%** | 56 | **94%** | **50%** | AR |  |
| 7 | *ABCC6* | c.1057G>A | p.Ala353Thr | **0.00%** | 58 | **93%** | **50%** | AR |  |
| 7 | *FBN1* | c.4283G>A | p.Arg1428His | **0.00%** | 29 | **100%** | **50%** | AD |  |
| 10 | *MED12** | c.3148C>T | p.Arg1050Cys | **0.00%** | **180** | **100%** | **50%** | XLD&XLR |  |
| 13 | *COL1A2* | c2077C>T | p.Arg693Trp | **0.00%** | **101** | **98%** | **100%** | AD&AR |  |
| 19 | *COL9A3* | c.1536C>T | p.Pro513Leu | **0.01%** | 98 | **98%** | **50%** | AD&AR |  |
| 24 | *COL12A1* | c.8501C>G | p.Pro2834Arg | **0.00%** | **103** | **100%** | **70%** | AD&AR |  |
| 29 | *COL2A1* | c.3047G>A | p.Arg1016Lys | **0.01%** | 26 | **100%** | **100%** | AD |  |
| 30 | *ATP6V0A2* | c.539T>C | p.lle180Thr | **0.01%** | 89 | **98%** | **100%** | AR |  |
| 35 | *EFEMP2* | c.773G>A | p.Cys258Tyr | **0.00%** | **194** | **100%** | **100%** | AR |  |
| 37 | *COL9A1* | c.2456C>T | p.Pro819Leu | **0.01%** | 98 | **100%** | **100%** | AD&AR |  |
| 41 | *RIN2* | c.77C>T | p.Ser26Leu | **0.01%** | **145** | **98%** | 0% | AR |  |
| 50 | *PKD1* | c.4835C>T | p.Thr1612Met | **0.01%** | 81 | **100%** | **60%** | AD |  |
| 50 | *TGFBR1* | c.854A>G | p.His285Arg | **0.00%** | 29 | **100%** | **50%** | AD |  |
| 59 | *COL11A2* | c.1615C>T | p.Arg539Trp | 0.12% | **101** | **100%** | **70%** | AD&AR |  |
| 68 | *TGFB3* | c.557T>G | p.Ile186Ser | **0.00%** | **142** | **95%** | **55%** | AD |  |
| 70 | *COL3A1* | c.2233G>A | p.Glu745Lys | **0.00%** | 56 | **95%** | **80%** | AD&AR |  |
| 72 | *EFEMP2* | c.460G>A | p.Gly154Ser | **0.00%** | 56 | **100%** | **50%** | AR |  |
| 75 | *CBS* | c.394C>T | p.Arg132Cys | **0.03%** | **180** | **93%** | 0% | AR |  |
| 75 | *COL9A3* | c.331C>T | p.Pro111Ser | **0.02%** | 74 | **98%** | **55%** | AD&AR |  |
| 79 | *TNXB* | c.9574G>A | p.Asp3192Asn | **0.01%** | 23 | **97%** | **50%** | AD&AR |  |
| 80 | *COL9A2* | c.818G>A | p.Pro273Arg | **0.00%** | **103** | **100%** | **70%** | AD |  |
| 90 | *COL12A1* | c.2378T>A | p.Ile793Asn | **0.00%** | **149** | **95%** | 36% | AD&AR |  |
| 91 | *B3GALT6* | c.593G>T | p.Trp198Leu | **0.00%** | 61 | **100%** | **67%** | AR |  |
| 91 | *COL1A2* | c.3055C>T | p.Leu1019Phe | **0.00%** | 22 | **95%** | **100%** | AD&AR |  |
| 91 | *FBN2* | c.2993T>A | p.Ile998Asn | **0.01%** | **149** | 87% | **89%** | AD |  |
| 91 | *TGFBR2* | c.4G>T | p.Gly2Cys | **0.01%** | **159** | **96%** | **50%** | AD |  |
| 103 | *LTBP4* | c.1417C>T | p.Arg473Cys | **0.01%** | **180** | N/A | **50%** | AR |  |
| 104 | *B3GALT6* | c.341C>A | p.Ala114Glu | **0.00%** | **107** | **90%** | 11% | AR |  |
| 105 | *ZNF469* | c.10967C>T | p.Ser3656Leu | **0.01%** | **145** | 57% | **50%** | AR |  |
| 106 | *PKD1* | c.10939C>T | p.Arg3647Trp | **0.02%** | **101** | **100%** | **60%** | AD |  |
| 109 | *COL1A1* | c.2386C>T | p.Arg796Cys | **0.00%** | **180** | **100%** | **100%** | AD |  |
| Included are details for the variants identified and reported by Fulgent Genetics Laboratory to be of unknown clinical significance and met at least three of the additional criteria provided in genetic testing indicating they were likely damage to the encoded protein product and considered in this study to be 'potentially pathogenic'. These criteria were defined as: 1) Maximum Allele Frequency ≤ 0.03% based on the gnomAD dataset at the time of genetic testing, 2) Grantham distance > 100 indicating substantial evolutionary distance between referent and substituted amino acids, 3) evolutionary conservation of amino acid across mammals ≥ 90%, and 4) at least half of the *in silico* predictions reported indicating the variant was deleterious. Criteria met by each variant are indicated in bold. The inheritance pattern(s) observed for causal variants in the gene are listed. AD=autosomal dominant, AR=autosomal recessive, XLD=X-linked dominant and XLR=X-linked recessive.  *denotes gene with X-linked, potentially dominant inheritance identified in female patient. | | | | | | | | | |
